# Bariatric surgery mitigated electrocardiographic abnormalities in patients with morbid obesity

**DOI:** 10.1038/s41598-024-57155-2

**Published:** 2024-03-20

**Authors:** Mehdi Bazrafshan, Soroush Nematollahi, Maliheh Kamali, Ariya Farrokhian, Nader Moeinvaziri, Hanieh Bazrafshan, Niusha Noormohammadi, Ali Mohammad Keshtvarz Hesam Abadi, Hamed Bazrafshan drissi

**Affiliations:** 1 Shiraz University of Medical Sciences, Shiraz, Iran; 2 Tehran Heart Center, Cardiovascular Diseases Research Institute, Tehran University of Medical Sciences, Tehran, Iran; 3 Department of Cardiology, Shiraz University of Medical Sciences, Shiraz, Iran; 4 Laparoscopy Research Center, Surgery Department, Shiraz University of Medical Sciences, Shiraz, Iran; 5 Clinical Neurology Research Center, Shiraz University of Medical Sciences, Shiraz, Iran; 6 Clinical Research Development Center, Nemazee Hospital, Shiraz University of Medical Sciences, Shiraz, Iran; 7 Cardiovascular Research Center, Shiraz University of Medical Sciences, Shiraz, Iran

**Keywords:** Cardiology, Obesity

## Abstract

Obesity can lead to cardiovascular dysfunctions and cause electrocardiographic disruptions. Bariatric surgery plays a significant role in weight loss. To assess its benefits, this study investigated electrocardiographic changes before and after bariatric surgery. The present article describes a retrospective cohort study with a 6-month follow-up period. Electrocardiograms were interpreted and compared before and six months after surgery. The relationships between weight loss, type of surgery, and electrocardiographic alterations were analyzed. A total of 200 patients participated in the study, with 34 (17%) men and 166 (83%) women. The mean age of the participants was 44.6 ± 8.6, and their mean body mass index was 43.8 ± 5.5 kg/m^2^. The mean of QTc decreased after the surgery, while the Sokolow-Lyon scores increased. The statistical analysis showed that QTc dispersion (> 40) (P < 0.001), right ventricular hypertrophy (P < 0.001), abnormal R wave progression (P < 0.001), QTc (P < 0.001) and Sokolow-Lyon criteria (P < 0.001) significantly changed postoperatively. In conclusion, bariatric surgery can reduce QTc, correct poor R wave progression, and resolve right ventricular hypertrophy (RVH) in patients with morbid obesity.

## Introduction

Obesity, the souvenir of modern lifestyle, has become a major universal concern in recent years. The prevalence of obesity has surpassed one billion worldwide. According to the World Health Organization (WHO), it is estimated that more than 167 million people worldwide will struggle with health issues due to obesity by 2025^1,2^. Obesity is defined as a body mass index (BMI) > 30 kg/m^2^ and can cause a greater risk of diabetes, hypertension, and hyperlipidemia, which results in more cardiovascular events^3^. A recent study reported that almost three million people die from obesity-induced cardiovascular diseases annually^4^. Furthermore, studies have shown that obesity affects cardiac electrophysiology directly by changing the heart’s structure^3,5,6^. Excess visceral adipocytes trigger the secretion of “adipokines”, which can lead to myocardial hypertrophy^3^. Hypertension secondary to obesity can also promote left ventricle hypertrophy (LVH); however, LVH is also observed in normotensive severely obese patients^7,8^. Cardiac morphologic changes can result in electrocardiographic alterations such as QTc dispersion, P-wave, and QTc prolongation^3^. The QT interval or corrected QT (QTc), an indicator of ventricular repolarization, has been reported to be prolonged in patients with obesity^9,10^. Studies have shown that long QTc is associated with fatal arrhythmias and sudden cardiac death^11,12^.

Moreover, changes in heart structure can lead to serious heart problems, such as atrial fibrillation (AF). Weight loss can significantly improve diabetes, dyslipidemia, and cardiovascular complications^13^. However, studies have shown that primary weight loss methods such as lifestyle modifications and dietary vigilance are ineffective in the long term. Some studies have shown that these techniques have limited long-term utility in maintaining weight loss and have no significant impact on the incidence of cardiovascular events. Therefore, surgical strategies for overcoming the medical and habitual methods are needed to address obesity-related complications^3,5,14,15^. Studies revealed that patients struggling with obesity succeeded in losing up to 60% of their extra body weight^16^. Several bariatric surgeries, such as sleeve gastrectomy and classic bypass, are commonly performed. These surgeries can lead to significant improvements in heart failure, coronary artery disease, and cardiac rhythm abnormalities^17–19^.

Our study aimed to evaluate the impact of bariatric surgeries on electrocardiography and to determine whether surgical weight loss methods are beneficial for correcting obesity-related problems.

## Results

### Demographics

The study included 200 patients, 166 (83%) of whom were female. The mean age of the population was 44.6 ± 8.6 years. Most participants were between 25 and 60 years of age; no one was younger than 25 years, and only two patients were above this range. The majority of the patients were severely obese (BMI > 40 kg/m^2^), and the mean BMI among the patients was 43.8 ± 5.5 kg/m^2^. Table 1 provides a detailed overview of the demographic and baseline characteristics of the participants.

**Table 1:** Demographic and baseline characteristics of the participants.

Parameter	Value
Gender, n (%)	
Male	34 (17)
Female	166 (83)
Age, mean ± SD	44.6 ± 8.6
25–45	101 (50.5)
45–60	97 (48.5)
> 60	2 (1)
BMI, Mean ± SD	43.8 ± 5.5
30–45	60 (30)
> 40	140 (70)
Type of surgery, n (%)	
Sleeve	92 (46)
Classic bypass	108 (54)

*BMI* body mass index.

### Electrocardiographic alterations

Table 2 shows the ECG changes observed before and after surgery. The table shows that bariatric procedures significantly improved R wave progression (60 versus 27, P < 0.001), corrected RVH (63 versus 18, P < 0.001), reduced QTc dispersion (139 versus 35, P < 0.001), shortened the QTc interval (443.9 versus 409.7, P < 0.001), and increased the Sokolow-Lyon score (15 versus 17.17, P < 0.001).

**Table 2:** Electrocardiographic changes. Preoperative vs. postoperative (6 months).

ECG changes	Preoperative	Postoperative	p value
The widened QRS complex, n (%)	1 (0.5)	1 (0.5)	–
Abnormal R wave progression, n (%)	60 (30)	27 (13.5)	< 0.001^†^
Right ventricle hypertrophy, n (%)	63 (31.5)	18 (9)	< 0.001^†^
ST depression, n (%)	1 (0.5)	0 (0)	–
Inverted T wave, n (%)	1 (0.5)	0 (0)	–
Premature ventricular contraction, n (%)	1 (0.5)	1 (0.5)	–
QTc dispersion > 40, n (%)	139 (69.5)	35 (17.5)	< 0.001^†^
QTc, Mean ± SD	443.9 ± 23.4	409.7 ± 20.5	< 0.001‡
Sokolow-Lyon score, Mean ± SD	15.0 ± 3.8	17.17 ± 3.16	< 0.001‡

*ECG* electrocardiography.

^†^Related-samples McNemar-test.

^‡^Paired-samples t-test.

### Correlations and regression analysis

We examined whether the demographic and anthropometric factors and the type of bariatric surgery impacted postoperative changes. We conducted a multiple regression analysis for QTc and the Sokolow-Lyon score. However, none of the dependent variables significantly affected either of these parameters. We used binary logistic regression to analyze the relationships between dichotomous variables such as QTc dispersion, RVH, and abnormal R wave progression. Only the type of bariatric procedure significantly predicted abnormal R wave progression (OR: 0.434, P = 0.025). Tables 3 and 4 demonstrate the results of the regression analysis in detail.

**Table 3:** The associations of sex, age, BMI, and surgery type with changes in the QTc and Sokolow-Lyon index were assessed via multiple regression analysis.

Coefficients	Dependent variables	Coefficient's p value	Standardized β coefficient	R^2^	df	F	Model's p value
QTc	Gender	0.148	0.104	0.017	4	0.828	0.509
	Age	0.426	0.057				
	BMI	0.669	0.031				
	Surgery type	0.390	0.062				
Sokolow-Lyon score	Gender	0.104	− 0.116	0.015	4	1.762	0.138
	Age	0.260	0.080				
	BMI	0.395	− 0.061				
	Surgery type	0.109	− 0.114				

*BMI* body mass index, *df* degrees of freedom.

**Table 4:** The effects of sex, age, BMI, and surgery type on QTc dispersion, RVH, and abnormal R wave progression after bariatric surgery according to binary logistic regression analysis.

Dependents	Coefficients	Coefficient's p value	Exp (B) (OR)	95% CI		R^2^	df	Chi-Square	Model's p value
				Lower	Upper				
QTc Dispersion	Gender	0.361	1.423	0.667	3.033	0.036	4	5.355	0.253
	Age	0.468	1.236	0.697	2.194				
	BMI	0.055	0.534	0.281	1.013				
	Surgery type	0.831	1.064	0.601	1.885				
RVH	Gender	0.917	1.048	0.435	2.523	0.019	4	2.556	0.635
	Age	0.358	0.732	0.376	1.424				
	BMI	0.244	1.574	0.734	3.376				
	Surgery type	0.951	0.980	0.506	1.895				
Abnormal R wave progression	Gender	0.163	2.249	0.720	7.030	0.085	4	10.619	0.031‡
	Age	0.144	0.579	0.278	1.205				
	BMI	0.287	0.658	0.304	1.432				
	Surgery type	0.025^†^	0.434	0.210	0.898				

*BMI* body mass index, *RVH* right ventricle hypertrophy, *OR* odds ratio, *CI* confidence interval, *df* degrees of freedom.

^†,‡^Chi-square of binary logistic regression.

## Discussion

Obesity is a well-known risk factor for various cardiac complications. Therefore, weight is an essential intervention in managing these conditions. Studies have shown that while any weight loss method can improve cardiac function and lower the risk of cardiovascular events^13^, surgical methods provide the best short-term and long-term benefits compared to pharmacological treatments and lifestyle modifications^3^.

In the current study, we aimed to evaluate the effects of bariatric surgery on ECG alterations during a 6-month follow-up period. The most common preoperative abnormality was QTc dispersion in 69.5% of participants, followed by RVH and abnormal R wave progression in 31.5% and 30% of the participants, respectively. However, all three abnormalities were significantly improved after bariatric surgery (P < 0.001). The mean QTc duration was 443.9 ± 23.4 ms, which markedly decreased to 409.7 ± 20.5 ms after the 6-month follow-up (P < 0.001). Our findings suggest that weight loss achieved through bariatric surgery significantly shortened the QTc duration, improved QTc dispersion and RVH and corrected R wave progression (Table 2). The Sokolow-Lyon criteria indicate LVH if the score exceeds 35. Our findings revealed that the score significantly increased after the surgery. This could be explained by the fact that after surgery, patients experienced weight loss, leading to less subcutaneous fat, better positioning of the heart in the thorax, and lower afterload pressure in the left ventricle, which can result in improved left ventricle function. However, although the Sokolow-Lyon score increased, none of the participants developed LVH within the 6-month follow-up period.

A regression analysis was conducted to predict ECG improvements based on sex, age, BMI, and type of surgery, as presented in Table 3. None of these variables had any statistically significant impact on the ECG. However, a statistically meaningful relationship was found between the type of surgery and abnormal R wave progression. Regression analysis revealed that sleeve gastrectomy was less effective than classic bypass in correcting abnormal R wave progression (OR:0.434, P = 0.025). The binomial logistic regression model was statistically significant (χ2(4):10.619; P = 0.031). The model explained 8.5% (Nagelkerke *R2*) of the variance in abnormal R wave progression and correctly classified 78.8% of the cases. The reason for these improvements lies in the hormonal and autonomic consequences of bariatric surgeries. Previous studies have shown that insulin resistance (IR) is one of the leading causes of prolonged QTc and QT dispersion^6,20–25^. Insulin has been found to hyperpolarize both excitable and nonexcitable cells, and can directly prolong the QT interval^26^. Excess insulin can indirectly cause QTc prolongation by inducing a hyperkalemic state, which is a known cause of this abnormality^27^. Moreover, higher doses and prolonged periods of hyperinsulinemia can result in increased release of catecholamines, leading to increased blood pressure. Over time, hypertension caused by elevated catecholamine release can cause left ventricle hypertrophy. Studies have suggested that an increased left ventricle mass is one of the most important causes of rhythm abnormalities, particularly QT prolongation^28,29^. Significant weight loss is the second most important cause of ECG changes after bariatric surgery. One study showed that individuals lose approximately 25%-30% of their initial weight or achieve 50%-70% excess weight loss after a successful surgery^30^. This substantial weight loss results in various cardiovascular changes^31^. These include a decrease in BMI, which can lead to lower blood pressure, reduced preload, and a decrease in ventricle mass in the long-term. Recent studies have shown that a prolonged QT interval is related to obstructive sleep apnea and sympathetic nervous system activation^5,7,14,32^. Losing weight by any means is expected to improve obstructive sleep apnea and reduce sympathetic activation, resulting in a normalized QT interval.

Our research findings support previously published articles on the effects of bariatric surgery on patients with obesity. Pontiroli et al.^33^ conducted a study on 116 patients with obesity who underwent bariatric surgery. They found that LVH and QT prolongation were common prior to surgery. However, one year after the surgery, the QTc decreased in all patients (P < 0.05), and LVH was resolved in the majority of the participants (P = 0.0025). In a similar study, Al-Salameh et al.^34^ assessed 28 patients who underwent sleeve gastrectomy and compared QTc changes three months after the operation. They discovered an average decrease of 28.46 ± 15.61 ms in the QTc within the first three months after the surgery (P < 0.0001). Notably, no statistically significant correlation was found between weight loss and decreased QTc^34^.

In a study conducted by Bezante et al.^31^ 85 individuals with severe obesity were examined in a case–control setting. Of these, 55 people underwent biliopancreatic diversion, while the remaining 30 were in the control group. The researchers found a significant reduction in QTc and QT dispersion after the operation (P < 0.0001). However, they discovered no correlation between weight loss percentage and QTc changes. They concluded that normalizing ventricular rhythm abnormalities is much more responsive to serum glucose and insulin levels than to absolute weight loss^31^.

We must acknowledge that our study had a few limitations to consider. First, we did not measure weight loss or postoperative BMI percentage in our study group. These measurements are crucial for understanding the correlation between the amount of weight loss and the QTc in the short term. Second, we need laboratory data such as glucose, insulin, and lipid profiles to make our study more comprehensive. By comparing these values, we could identify further correlations and insights.

Finally, we conclude that bariatric surgeries can correct abnormal R wave progression and resolve QT dispersion in morbidly obese patients. The QTc interval significantly decreased within six months postoperatively. Given the positive impact on the cardiovascular system, bariatric surgery can be a practical and beneficial choice for morbidly obese patients who are struggling with cardiac complications.

## Methods

### Ethics statement

The study was carried out in accordance with the principles of the Declaration of Helsinki. All authors confirm that all methods were performed in accordance with the relevant guidelines and regulations. The study started after approval from the Shiraz University of Medical Sciences Ethics Committee (approval ID: IR.SUMS.MED.REC.1401.056, approval date: 2022.05.01). The patients undergoing bariatric surgery had to provide written informed consent that informed them of the potential risks during and after the surgery. Patients agreed to participate in a long-term follow-up after the surgery.

### Population and study design

**Figure 1:**
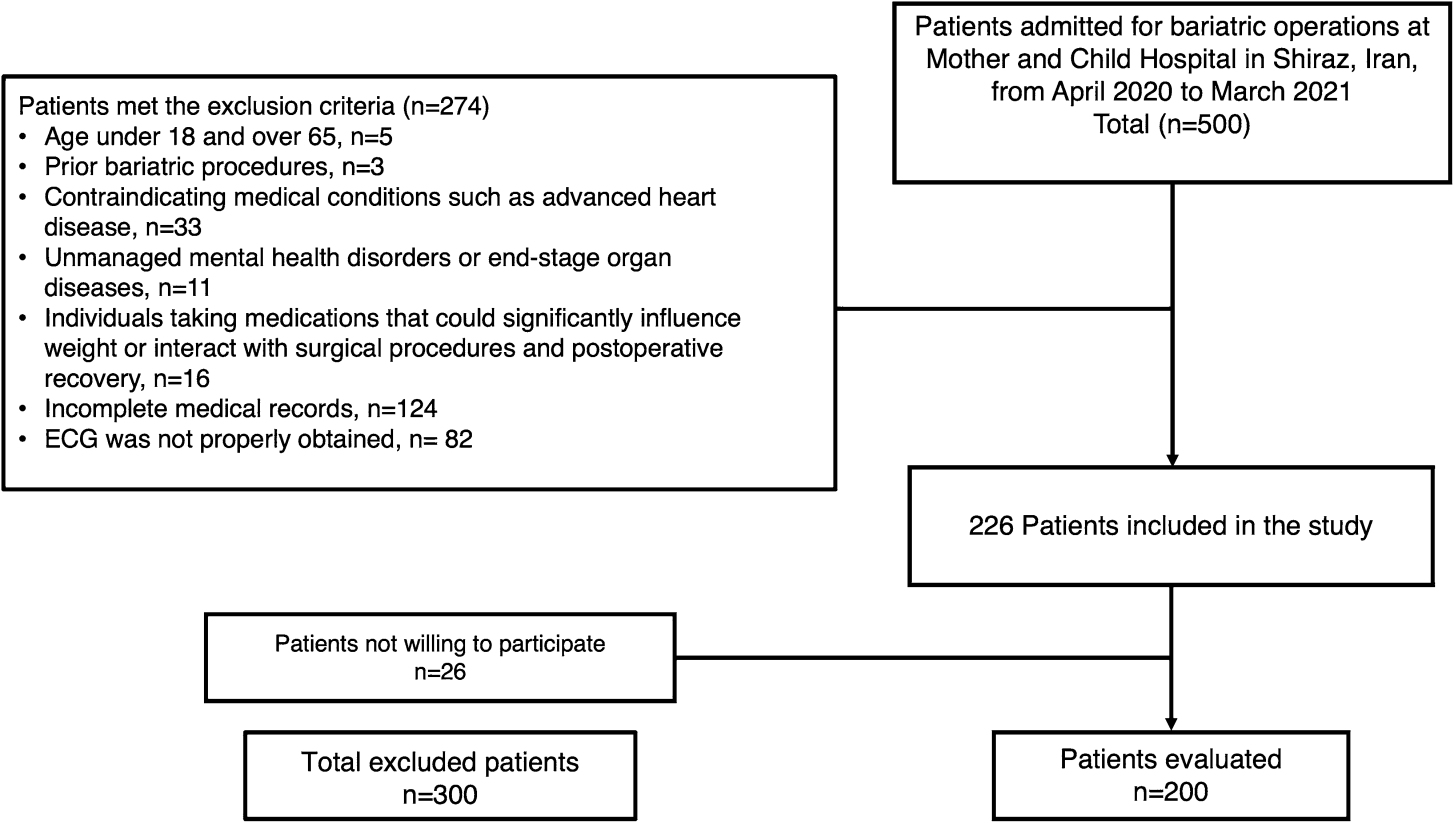
The flowchart of patient selection process.

In the current cohort, we included 200 patients who underwent bariatric surgery at Mother and Child Hospital in Shiraz, Iran, from April 2020 to March 2021. We used the census method to determine the study population size and enrolled 500 patients admitted for bariatric operations. Ultimately, 200 patients met the inclusion criteria: were candidates for bariatric surgery, had complete medical records, and provided informed consent. Patients who did not consent to participate or had incomplete medical records were excluded from the survey. The patient selection, exclusions, and inclusions are shown in Fig. 1.

We collected demographic information, past medical histories, and intraoperative and postoperative data from the patients' medical records using a prepared checklist. The criteria for bariatric surgeries include the following:

1. BMI > 40 kg/m^2^,
2. BMI > 35 kg/m^2^ accompanied by a significant risk factor, e.g., diabetes and hypertension,
3. Poor response to primary weight loss methods, such as exercise, diet, and medical approaches.

Exclusion criteria were considered to remove variables that may bias the results and hamper a clear safety assessment for both patients and researchers. The following exclusion criteria were then applied (Fig. 1):

1. Aged under 18 and over 65,
2. Prior to bariatric procedures,
3. Loss to follow-ups,
4. Contraindicating medical conditions such as advanced heart disease, unmanaged mental health disorders, or end-stage organ diseases,
5. Individuals who reported medical interference, who were taking medications that could significantly influence weight or interact with surgical procedures or who experienced postoperative recovery were excluded.

The patients were then divided into two groups based on the type of bariatric surgery they received: sleeve gastrectomy and classic bypass. An electrocardiogram was obtained before the surgery and six months postoperatively. An expert cardiologist blindly interpreted and compared the ECGs before and after surgery.

### Statistical analysis

Continuous variables are reported as the mean ± SD, while quantitative values are reported as the frequency. To check normality, we used Shapiro–Wilk and Kolmogorov–Smirnov tests and related histograms. We used a paired t-test to compare the means for normally distributed variables, while nonparametric tests were performed for nonnormally distributed values. To analyze categorical data, we conducted a chi-square test. We also ran multiple and binary logistic regression analyses to predict possible correlations between variables. We considered p-values less than 0.05 to indicate statistical significance. The data were analyzed using SPSS version 23.

## Data Availability

The ethnical appendix, statistical code, and dataset are available based on requests sent to the corresponding author.
