# Author Correction: Bariatric surgery mitigated electrocardiographic abnormalities in patients with morbid obesity

**DOI:** 10.1038/s41598-024-70964-9

**Published:** 2024-08-27

**Authors:** Mehdi Bazrafshan, Soroush Nematollahi, Maliheh Kamali, Ariya Farrokhian, Nader Moeinvaziri, Hanieh Bazrafshan, Niusha Noormohammadi, Ali Mohammad Keshtvarz Hesam Abadi, Hamed Bazrafshan drissi

**Affiliations:** 1 Shiraz University of Medical Sciences, Shiraz, Iran; 2 Tehran Heart Center, Cardiovascular Diseases Research Institute, Tehran University of Medical Sciences, Tehran, Iran; 3 Department of Cardiology, Shiraz University of Medical Sciences, Shiraz, Iran; 4 Laparoscopy Research Center, Surgery Department, Shiraz University of Medical Sciences, Shiraz, Iran; 5 Clinical Neurology Research Center, Shiraz University of Medical Sciences, Shiraz, Iran; 6 Clinical Research Development Center, Nemazee Hospital, Shiraz University of Medical Sciences, Shiraz, Iran; 7 Cardiovascular Research Center, Shiraz University of Medical Sciences, Shiraz, Iran

Correction to: *Scientific Reports* 10.1038/s41598-024-57155-2, published online: 20 March 2024

The original version of this Article contained an error in the spelling of the author Ali Mohammad Keshtvarz Hesam Abadi which was incorrectly given as Mohammad Keshtvarz Hesam Abadi.

The original Article has been corrected.

